# Supplementary material for: Hydrothermal Synthesis of Bismuth Ferrite Hollow Spheres with Enhanced Visible-Light Photocatalytic Activity
Source: Molecules. 2023 Jun 29;28(13):5079. doi: 10.3390/molecules28135079 (PMC10343919; doi:10.3390/molecules28135079)
Supplement: Supplementary file 1 [file molecules-28-05079-s001.zip › molecules-2455776-supplementary.pdf]

Hydrothermal Synthesis Of Bismuth Ferrite Hollow Spheres With Enhanced Visible-Light Photocatalytic Activity

Thomas Cadenbach [1,*], Valeria Sanchez [2], Daniela Chiquito Rios,[1] Alexis Debut [3], Karla Vizuete [3] and Maria J. Benitez [2,*]
1 Colegio de Ciencias e Ingenierías, Universidad San Francisco de Quito, Diego de Robles y Vía Interoceánica, Quito 170901, Ecuador

2 Departamento de Física, Facultad de Ciencias, Escuela Politécnica Nacional, Ladrón de Guevara E11-253, Quito 170525, Ecuador

3 Centro de Nanociencia y Nanotecnología, Universidad de las Fuerzas Armadas ESPE, Av. Gral. Rumiñahui s/n, Sangolquí 171103, Ecuador; apdebut@espe.edu.ec (A.D.); ksvizuete@espe.edu.ec (K.V.)

* Correspondence: tcadenbach@usfq.edu.ec (T.C.); maria.benitezr@epn.edu.ec (M.J.B.)

Supporting Information

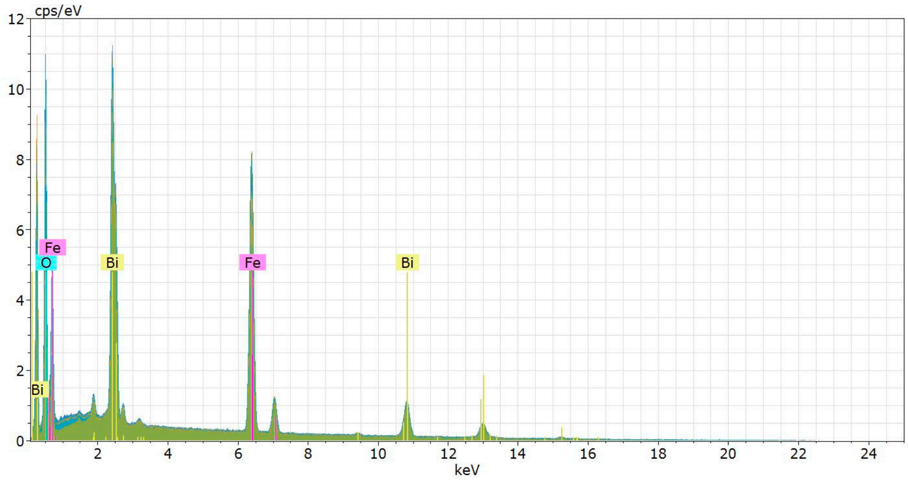

Atom	Theoretical Subtotal Mass (%)	Measured
Bi	66.81	67.59
Fe	17.85	17.21
O	15.34	15.74

Figure S1: EDS Spectrum of $BiFeO_3$ Hollow Spheres.

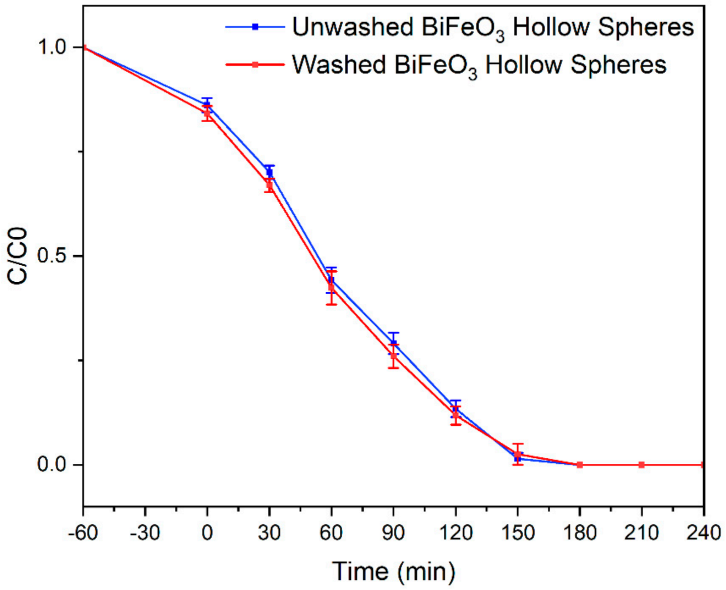

Figure S2. Removal of Rhodamine B as a function of irradiation time under visible-light using unwashed and washed $BiFeO_3$ hollow spheres

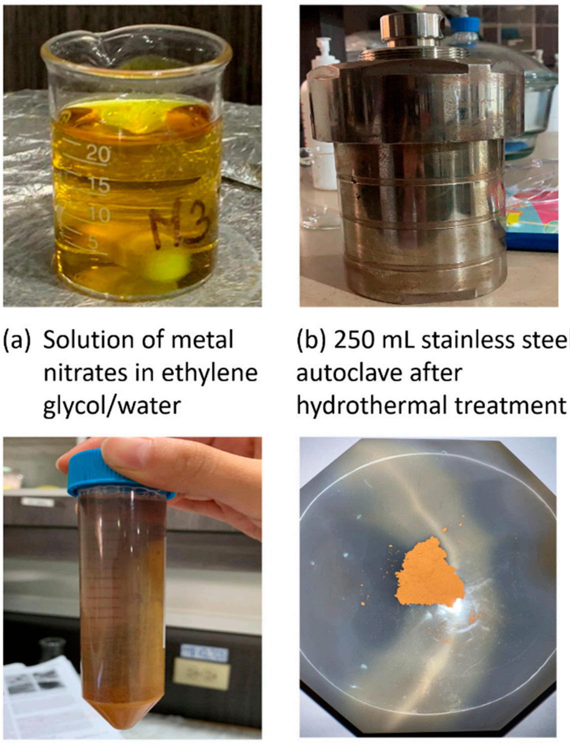

(a) Solution of metal nitrates in ethylene glycol/water

(b) 250 mL stainless steel autoclave after hydrothermal treatment

(c) Reaction mixture prior to centrifugation

(d) Sample after calcination

Figure S3. Schematic representation of experimental methods

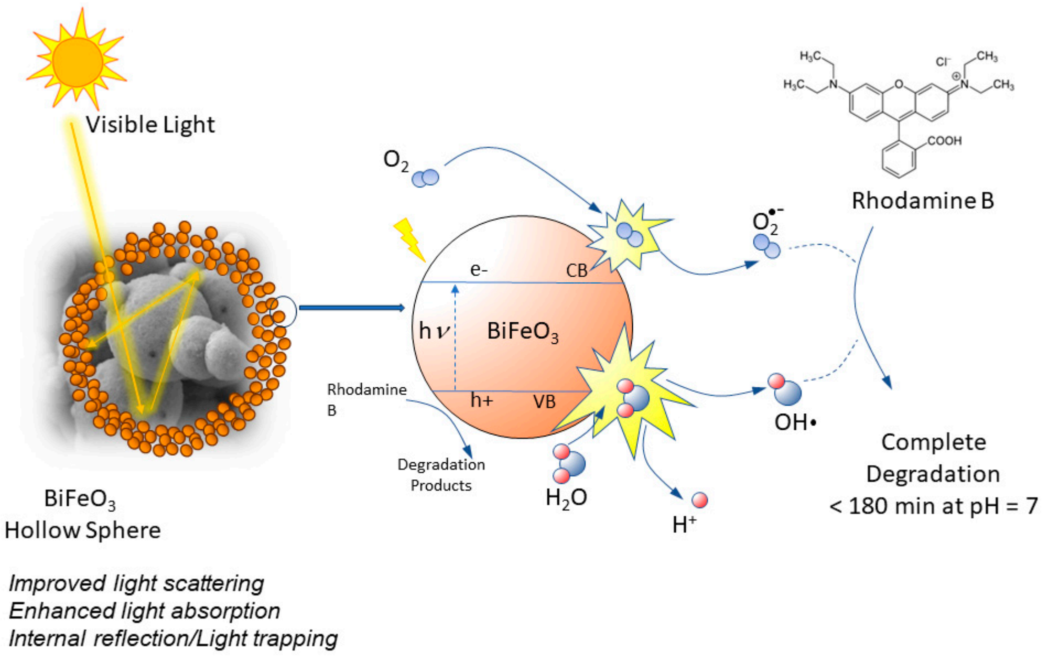

$BiFeO_3$ hollow spheres + $h\nu \rightarrow e^- + h^+$

$h^+ + H_2O \rightarrow \cdot OH$

h^+ + Rhodamine B $\rightarrow$ degradation products

$\cdot OH$ + Rhodamine B $\rightarrow$ degradation products

$\cdot O_2^-$ + Rhodamine B $\rightarrow$ degradation products

Figure S4. Photocatalytic mechanism of Rhodamine degradation using $BiFeO_3$ hollow spheres
